# The Molecular Mechanisms Underlying Zucchini-Induced Changes in the Host Adaptation of Cotton- and Cucumber-Type *Aphis gossypii*

**DOI:** 10.3390/biom15060791

**Published:** 2025-05-29

**Authors:** Yibin Pan, Weili Xu, Li Wang, Kaixin Zhang, Jichao Ji, Dongyang Li, Xiangzhen Zhu, Xueke Gao, Junyu Luo, Jinjie Cui

**Affiliations:** 1 Research Base of Zhengzhou University, State Key Laboratory of Cotton Bio-breeding and Integrated Utilization, Institute of Cotton Research, Chinese Academy of Agricultural Sciences, Anyang 455000, China; m13432317386@163.com (Y.P.); xwl18135725739@163.com (W.X.); wangli@caas.cn (L.W.); zhangkaixin@caas.cn (K.Z.); jijichao@caas.cn (J.J.); lidongyang@caas.cn (D.L.); luojunyu@caas.cn (J.L.); cuijinjie@caas.cn (J.C.); 2 College of Life Sciences, South China Agricultural University, Guangzhou 510642, China

**Keywords:** cotton aphid, host specialization, host shift, biological phenotype, combined transcriptome analysis

## Abstract

The polyphagous aphid *Aphis gossypii* exhibits host-specific biotypes, notably the cotton (Hap1) and cucumber (Hap3) types. While both can adapt to new hosts via zucchini induction, the underlying molecular mechanisms remain unclear. Our investigation revealed that both Hap1 and Hap3 *A. gossypii* underwent significant body size enlargement following host transfer to zucchini. Transcriptomic analysis revealed that zucchini-mediated host adaptation in the *A. gossypii* biotypes (Hap1/Hap3) involves insulin metabolism and detoxification pathways, with 17 co-differentially expressed genes (e.g., *Col-I* (type I collagen), *CYP450 6a13*, *peroxidase*) potentially critical for adaptation. The findings provide new insights into the molecular mechanisms regulating *A. gossypii* phenotypic plasticity and contribute to the development of resistance management strategies.

## 1. Introduction

Through an extended co-evolutionary process, phytophagous insects and their host plants have established relatively stable ecological symbiotic relationships [1]. Phytophagous insects rely on host plants for essential nutrients, which are necessary for their physiological processes and life cycle completion. However, due to inherent variations in nutrient composition, different host plants significantly influence the growth and development of these insects. Conversely, phytophagous insects exhibit preferences for specific host types, a phenomenon known as insect host specialization, which is of considerable biological importance [2]. A well-documented example is the peach aphid (*Myzus persicae*), which demonstrates substantial differences in survival across different hosts. It exhibits the highest survival rate and the longest body length on tobacco, whereas its survival rate is relatively low on peach leaves, its original host [3].

The cotton aphid (*Aphis gossypii*), a member of the Aphididae family, has an extraordinarily broad host range, feeding on over 900 plant species, including cotton, cucumber, and zucchini, among others [4]. Using its piercing–sucking mouthparts, it extracts sap from the plants, thereby disrupting their normal growth and development [5]. As a polyphagous insect, *A. gossypii* has evolved preferential feeding behaviors toward specific plant species over time, leading to the differentiation of distinct host specialization types [6]. The development of host-specific phenotypes in *A. gossypii* is a highly complex and multifaceted process, including genetic, physiological, and behavioral factors, as well as interactions with host plants [7]. From the host–plant perspective, key influencing factors include the physical and chemical properties of the plant surface, nutrient composition, and secondary metabolites. From the aphid’s perspective, olfactory and taste receptors, detoxification metabolism, and immune responses play crucial roles in host specialization. These factors interact dynamically, ultimately shaping the host specificity of *A. gossypii*.

The selective localization of *A. gossypii* on host plants represents a crucial initial step in the formation of specialized phenotypes. Before selecting a host, *A. gossypii* evaluates plant suitability based on microscopic traits such as trichome morphology, waxy layer thickness, and surface texture [8]. Simultaneously, *A. gossypii* engage in brief probing behavior to assess feeding ease. The nutritional components of the host plant, along with chemical factors such as stimulants and inhibitors, significantly influence the development of specialized phenotypes [9]. The types and concentrations of free amino acids in host plants are likewise significant factors in aphid growth and reproduction, potentially impacting host specialization [10].

During aphid feeding, host plants generally synthesize and accumulate various secondary metabolites as defense mechanisms, which can be toxic to insects. However, aphids possess a highly developed detoxification enzyme system capable of metabolizing these plant defense compounds, thereby reducing or neutralizing their toxic effects. Research shows that insects develop host specialization largely through adaptations in their detoxification systems [11]. For example, the host adaptation process in the green peach aphid (*Myzus persicae*) is mediated by the transcriptional activation of distinct detoxification gene networks (e.g., *CYP6CY3*, *GSTs*) [12,13]. Similarly, In Russian wheat aphids (*Diuraphis noxia*), the RWA1 and RWA2 biotypes produce different isoforms of the detoxification enzyme glucose dehydrogenase through evolution [14,15].

Traditional bioassay studies classify *A. gossypii* into host-specific groups, including those associated with hibiscus, miscanthus, chrysanthemum, cucumber, taro, eggplant, cotton, and potato [16,17]. *A. gossypii* exhibit poor performance on non-optimal hosts, characterized by high mortality and reduced fecundity. For example, Dutch researchers found that *A. gossypii* adapted to chrysanthemums and cucumbers nearly lost their ability to reproduce when transferred between these hosts [18]. Similarly, Satar et al. (2013) observed that *A. gossypii* failed to survive on citrus, cucumber, or okra [19]. Agarwala et al. (2013) demonstrated that *A. gossypii* specialized for taro and eggplant could not survive when transferred to each other’s hosts, based on host transfer tests and life table analysis [16]. Notably, although cotton- and cucumber-adapted aphids exhibit distinct host adaptations and cannot directly switch between these hosts, zucchini can serve as an intermediate plant. After feeding on zucchini for three generations, these aphids exhibit significantly enhanced adaptability to host switching [20]. This suggests that host specialization in *A. gossypii* is not fixed and may be modulated through physiological, biochemical, and transcriptomic adjustments.

Although previous studies have documented zucchini-induced changes in host adaptation among different *A. gossypii* biotypes, the underlying mechanisms remain poorly understood. Investigating these mechanisms is necessary to understand the evolutionary history of *A. gossypii* and aphid host adaptation more broadly. Therefore, this study aims to reveal the molecular basis of host adaptation shifts in Hap1 (cotton-type) and Hap3 (cucumber-type) *A. gossypii* following zucchini exposure. Specifically, we examined changes in body size across three consecutive generations after transfer to zucchini, combined with transcriptomic sequencing to analyze host specialization and adaptation mechanisms at the molecular level. The findings of this study provide a foundation for phenotypic regulation and resistance management in *A. gossypii*, contributing to the development of more effective pest management strategies.

## 2. Materials and Methods

### 2.1. Experimental Protocol

This study investigated host adaptation in *A. gossypii* through a three-phase approach, as follows: (1) host adaptation assays where Hap1 (cotton-type) and Hap3 (cucumber-type) aphids were transferred to zucchini leaves across three generations (T0-T2), with daily body size measurements (length/width) taken using ZEISS microscopy; (2) RNA-seq profiling of 3rd instar nymphs from original hosts (CKs) and zucchini-adapted generations, with RNA extracted using RNAiso Plus and sequenced on BGISEQ-500; (3) bioinformatics analysis including HISAT2 alignment, DEG identification (|log2FC| ≥ 1, *p* ≤ 0.05 via DEGseq), and functional annotation using the KOBAS (version 3.0)/InterProScan (version 5.50-84.0).

### 2.2. Insects and Plants

The *A. gossypii* used in this study were collected from cotton and cucumber plants in the east field of the experimental field at the Cotton Research Institute of the Chinese Academy of Agricultural Sciences (Anyang City, Henan Province). These aphids were then transferred to their respective host plants and maintained in an artificial intelligence incubator under controlled conditions (26 ± 1 °C, L:D = 14:10 h, RH = 70% ± 5%). DNA molecular identification was performed to distinguish between cotton-type (Hap1) and cucumber-type (Hap3) aphids. Both aphid types were reared in the laboratory for over 50 generations, during which they were not exposed to pesticides to ensure reliable experimental results.

The test plants consisted of three host species: cotton (*Gossypium hirsutum*, cultivar “Cotton Institute of China 49”), zucchini (*Cucurbita pepo*, cultivar “Chun Bai Yu”), and cucumber (*Cucumis sativus*, cultivar “Xin Jin You 1”). Cotton seeds were provided by the National Intermediate Cotton Germplasm Resources Bank, while cucumber and zucchini seeds were purchased from local suppliers. All test plants were cultivated in a greenhouse under controlled environmental conditions (26 ± 1 °C, RH = 70–80%, L:D = 14:10 h).

### 2.3. Changes in the Body Length and Width of Hap1 and Hap3 A. gossypii on Their Original Hosts and After Transfer to Zucchini

Experiments were conducted in an artificial intelligence light incubator under the following conditions: temperature = 26 ± 1 °C, humidity = 70 ± 5%, and photoperiod = L:D = 14:10 h. Leaves from cotton and cucumber plants were affixed to Petri dishes containing 1.8% agar by mass fraction, with fresh leaves replaced every 2–3 days to maintain a suitable environment for normal aphid growth.

For both Hap1 and Hap3 *A. gossypii*, 1st instar nymphs that had hatched within 12 h on their original host were transferred to Petri dishes containing leaves of their respective host plants and used as controls (CKs). Concurrently, multiple wingless adult Hap1 and Hap3 aphids from their original hosts were transferred to Petri dishes containing zucchini leaves. The nymphs produced by these adults within 12 h were designated as the T0 generation. Similarly, nymphs produced by the T0 generation were labeled as the T1 generation, while those produced by the T1 generation were recorded as the T2 generation.

Each aphid was treated as an independent sample, with twelve replicates per treatment. Body length and width were measured daily using a ZEISS Discovery.V8 SteREO microscope (Carl Zeiss AG, Jena, Germany) equipped with ZEN software (version 2.3) until the aphids’ death. Any offspring produced by adult aphids during this period were removed to prevent interference with measurements.

### 2.4. RNA-Seq Sample Preparation

To investigate transcriptomic changes in Hap1 and Hap3 *A. gossypii* following feeding on zucchini, 3rd instar aphids that had exclusively fed on cotton (Hap1) or cucumber (Hap3) were collected as CKs. The treatment group consisted of 3rd instar Hap1 and Hap3 aphids from the T0–T2 generations that had fed on zucchini. Three biological replicates were established for each treatment, with a minimum of 200 aphids per replicate.

Immediately after collection, all aphid samples were flash-frozen in liquid nitrogen and stored at −80 °C. The total RNA was extracted using the RNAiso Plus kit (a Takara Bio Company, Mountain View, CA, USA), following the manufacturer’s protocol. RNA purity and concentration were assessed using a NanoDrop 2000 spectrophotometer, (Beijing Labtech Instruments Co., Ltd., Beijing, China) while RNA integrity was verified via 1.2% agarose gel electrophoresis. High-quality RNA samples were used for cDNA synthesis and library construction. Illumina sequencing libraries were prepared by Wuhan Huada Science and Technology (BGI) Co. Ltd., using the DNBSEQ Transcriptome method, and sequencing was performed on the BGISEQ-500 platform.

### 2.5. Processing, Assembly, and Functional Annotation of Transcriptome Data

The raw transcriptome sequencing data generated by UWTSD Wuhan underwent quality control processing to remove low-quality reads. Clean reads were aligned to the *A. gossypii* reference genome using HISAT2, and transcript reconstruction was performed with StringTie and Cufflinks to identify novel transcripts. The gene expression levels were quantified using Bowtie2 (version 2.2.5) and RESM (version 1.2.8), and the fragments per kilobase per million reads (FPKMs) method was used to normalize the expression data. Differentially expressed genes (DEGs) were identified using DEGseq, which employs a Poisson distribution-based approach [21]. Genes with an absolute *p*-value ≤ 0.05 and |log2(Fold Change)| ≥ 1 were considered differentially expressed. The functional annotation of DEGs was performed using multiple bioinformatics tools. KEGG pathway analysis was conducted using the KEGG Orthology-Based Annotation System (KOBAS), while Gene Ontology (GO) homology results were obtained via InterProScan, which integrates multiple protein domain databases. Additionally, the amino acid sequences of specific genes were predicted and compared against the Pfam database using HMMER software (version 3.0) to obtain functional annotation information.

The functional annotation of DEGs was performed using multiple bioinformatics tools. KEGG pathway analysis was conducted using the KEGG Orthology-Based Annotation System (KOBAS), while Gene Ontology (GO) homology results were obtained via an InterProScan, which integrates multiple protein domain databases. Additionally, the amino acid sequences of specific genes were predicted and compared against the Pfam database using HMMER software to obtain functional annotation information.

### 2.6. Statistical Analysis

All statistical analyses were performed using IBM SPPS Statistics 22, while OriginPro 2021 and GraphPad Prism 9.5.1 software were used for data visualization. Data are presented as the mean ± SEM. Statistical significance was determined using one-way ANOVA followed by Tukey’s test (ns, *p* ≥ 0.05; * *p* < 0.05; **, *p* < 0.01), and for multiple comparisons, Fisher’s least significant difference (LSD) tests were conducted at the 0.05 significance level.

## 3. Results

### 3.1. Changes in Body Length and Width

**Figure 1:**
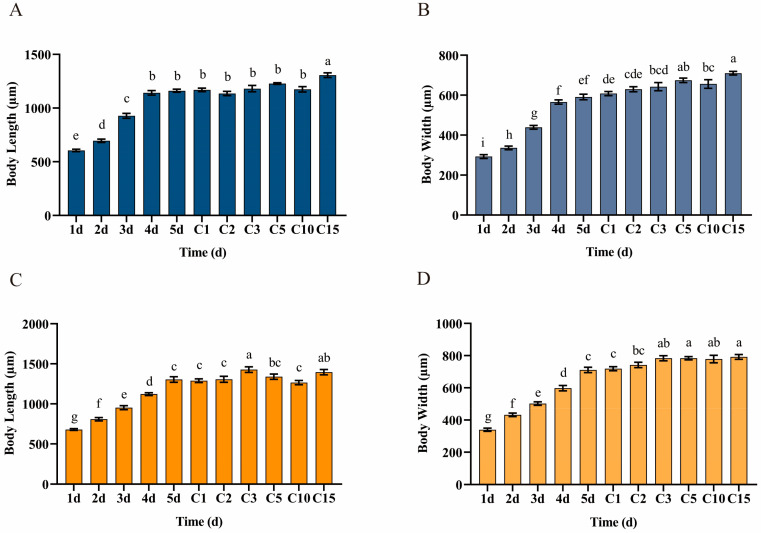
Dynamics of body length and body width in Hap1 and Hap3 *A. gossypii* throughout their growth and development cycle. (**A**) Body length variation of the Hap1 *A. gossypii* on cotton. (**B**) Body width variation of the Hap1 *A. gossypii* on cotton. (**C**) Body length variation of the Hap3 *A. gossypii* on cucumber. (**D**) Body width variation of the Hap3 *A. gossypii* on cucumber. Developmental stages are denoted as c1 (1 day after adulthood, DAA), c2 (2 DAA), c3 (3 DAA), c5 (5 DAA), c10 (10 DAA), and c15 (15 DAA). Data are means ± SEM, different letters indicate significance at the *p* <  0.05 level, as determined by one-way ANOVA following LSD test for multiple comparisons.

The data demonstrated a gradual increase in the body length and width of both Hap1 and Hap3 *A. gossypii* during their developmental stages from 1st instar to adulthood, stabilizing upon reaching the adult stage (Figure 1). Throughout development, Hap3 *A. gossypii* consistently exhibited larger body sizes compared to Hap1, with the body length and width increasing by 0.03–0.14 mm and 0.02–0.24 mm, respectively, relative to Hap1 *A. gossypii*.

**Figure 2:**
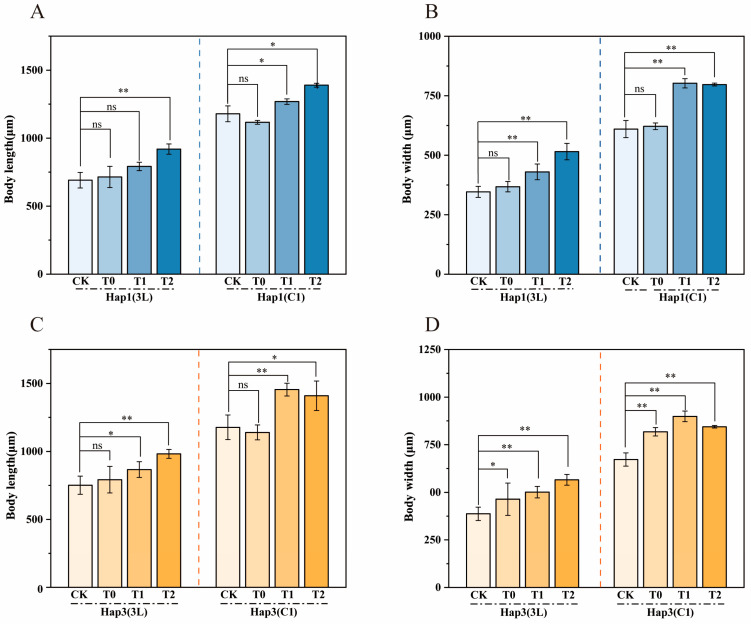
Alterations in body length and width of Hap1 and Hap3 *A. gossypii* after transfer to zucchini hosts in the T0-T2 generation at 3rd instar (3L) and one day after adulthood (C1). (**A**) Changes in body length of the T0-T2 generation after transfer of Hap1 *A. gossypii* to courgette. (**B**) Changes in body width of the T0-T2 generation after transfer of Hap1 *A. gossypii* to courgette. (**C**) Changes in body length of the T0-T2 generation after transfer of Hap3 *A. gossypii* to courgette. (**D**) Changes in body width of the T0-T2 generation after transfer of Hap3 *A. gossypii* to courgette. Data are means ± SEM, Tukey’s test, ns, *p* ≥ 0.05; * *p* < 0.05; **, *p* < 0.01.

The host plant species had a substantial impact on aphid body size. When Hap1 and Hap3 aphids were reared on zucchini across the T0 and T2 generations, an overall increase in body size was observed for both groups (Figure 2). Specifically, at the 3rd instar stage, the mean increases in body length and width for Hap1T0-T2 aphids were 0.02–0.23 mm and 0.02–0.17 mm, respectively, while Hap3T0-T2 aphids exhibited increases of 0.05–0.24 mm and 0.08–0.18 mm, respectively, compared to the CK. One day after reaching adulthood, the mean changes in body length and width were −0.06–0.21 mm and 0.01–0.19 mm for Hap1T0-T2 aphids and −0.04–0.28 mm and 0.15–0.23 mm for Hap3T0-T2 aphids compared to CK. Notably, the body size increase was more pronounced in Hap3 aphids than in Hap1 aphids when reared on zucchini, suggesting a greater adaptation capacity of Hap3 aphids to zucchini as a new host plant.

### 3.2. Transcriptome Analysis of the T0-T2 Generation of Hap1 A. gossypii Transferred to Zucchini

**Figure 3:**
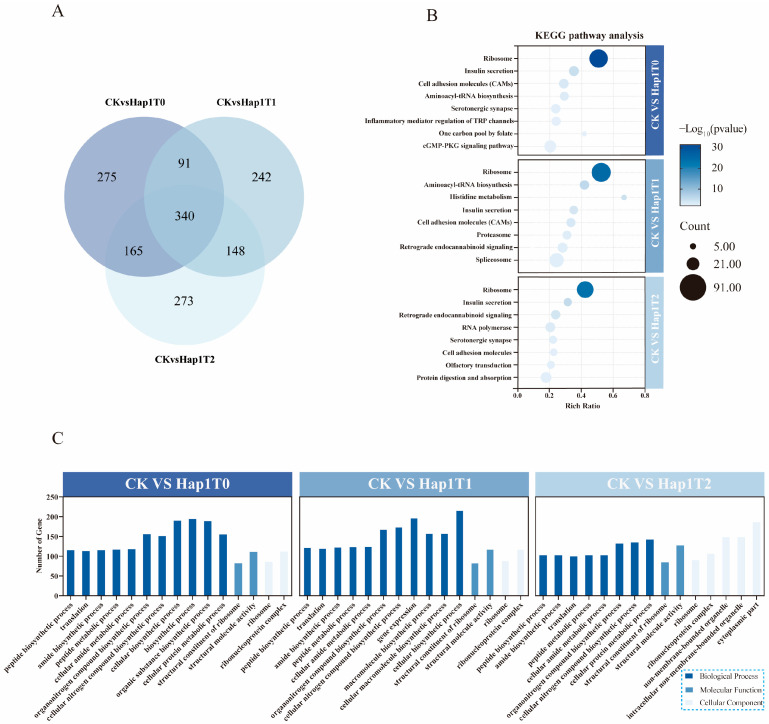
Joint Veen analysis showing the total number of DEGs in the T0-T2 generation of Hap1 *A. gossypii* (**A**); Top 8 KEGG pathways enriched with DEGs in the T0-T2 generation of Hap1 *A. gossypii* transferred to zucchini hosts (**B**); GO functional classification of DEGs in the T0-T2 generations after transferring Hap1 *A. gossypii* to zucchini hosts, highlighting the top 15 pathways (**C**).

Transcriptome analysis between the control group (CK; Hap1 aphids reared on cotton) and the experimental group (Hap1 aphids transferred to zucchini) revealed significant changes in gene expression. A total of 871, 821, and 926 DEGs were identified in the T0, T1, and T2 generations, respectively, with 340 DEGs exhibiting co-expression across all three generations (Figure 3A).

The functional annotation of DEGs using GO analysis classified them into three major categories: Biological Processes (BPs), Cellular Components (CCs), and Molecular Functions (MFs) (Figure 3C). BP-related DEGs were predominantly enriched in peptide biosynthesis, translation, amide biosynthesis, peptide metabolism, and the cellular nitrogen compound biosynthetic process. At the MF level, DEGs are predominantly enriched in the structural constituent of ribosome and structural molecular activity. In the CC category, significant enrichment was observed in ribosomal and ribonucleoprotein complexes.

KEGG pathway enrichment analysis identified the top eight metabolic pathways associated with DEGs (Figure 3B). In the T0 generation, DEGs were mainly enriched in the insulin secretion pathway, cell adhesion molecules (CAMs), aminoacyl-tRNA biosynthesis, and serotonergic synapse pathways. The T1 generation exhibited enrichment in insulin secretion, aminoacyl tRNA biosynthesis, histidine metabolism, cell adhesion molecules (CAMs), and proteasome pathways. Meanwhile, the T2 generation DEGs were predominantly enriched in the insulin secretion, RNA polymerase, and serotonergic synapse pathways. Notably, the ribosome synthesis and insulin secretion pathways were consistently enriched across all three generations, suggesting their critical role in Hap1 aphid adaptation to zucchini.

Further analysis revealed the significant upregulation of the *IGF-2* (insulin-like growth factor 2) (Log2FC Hap1T0: 1.15; Log2FC Hap1T1: 0.94; Log2FC Hap1T2: 1.03; *p* ≤ 0.05) in Hap1 aphids after transfer to zucchini. The results suggest that this gene may play a key role in the physiological adaptation of Hap1 *A. gossypii* to a new host environment.

### 3.3. Transcriptome Analysis of the T0-T2 Generations of Hap3 A. gossypii Transfer to Zucchini

**Figure 4:**
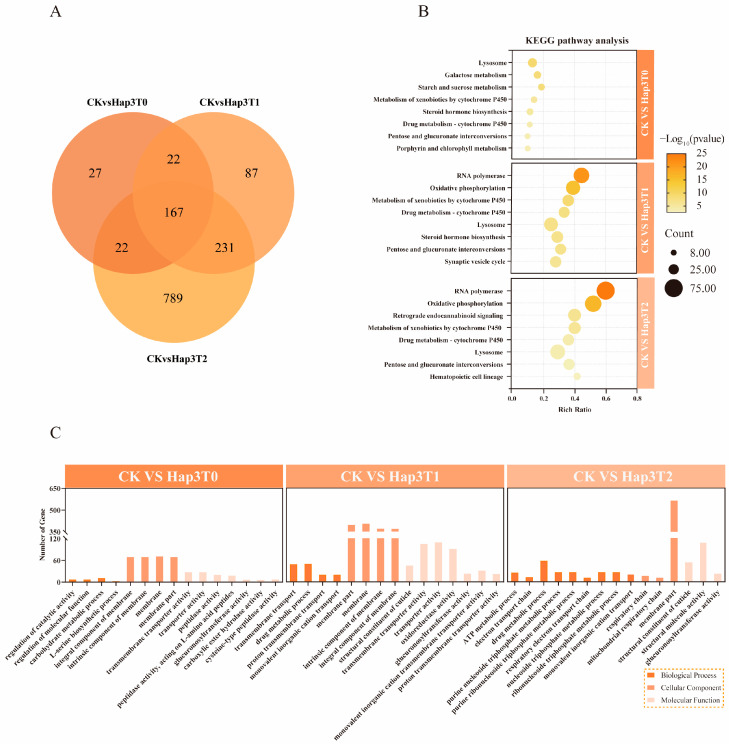
Joint Veen analysis showing the total number of DEGs in the T0-T2 generation of Hap3 *A. gossypii* (**A**); Top 8 KEGG pathways enriched with DEGs in the T0-T2 generation of Hap3 *A. gossypii* transferred to zucchini hosts (**B**); GO functional classification of DEGs in the T0-T2 generation after transferring Hap3 *A. gossypii* to zucchini hosts, highlighting the top 15 pathways (**C**).

To investigate the molecular mechanisms underlying the host adaptation of cucumber-type Hap3 *A. gossypii* induced by an intermediate host, transcriptomic analyses were conducted on Hap3 *A. gossypii* transferred from their original host to zucchini across the T0 to T2 generations. Compared with the CK, 238, 507, and 1,209 DEGs were identified in the T0, T1, and T2 generations, respectively (Figure 4A). Further analysis revealed that 167 DEGs exhibited a cross-generational co-expression pattern between the T0 and T2 generations.

GO enrichment analysis of DEGs in the T0 to T2 generations indicated that, in the BP category, DEGs were primarily enriched in ATP metabolic processes, drug metabolic processes, and the regulation of molecular functions. In the CC category, DEGs were mainly distributed in the intrinsic components of the cell membrane and on the cell membrane. At the MF level, DEGs were primarily associated with glucuronosyltransferase activity, oxidoreductase activity, transporter activity, and transmembrane transporter protein activity (Figure 4C).

KEGG pathway enrichment analysis revealed the top eight metabolic pathways containing the highest number of DEGs (Figure 4B). In the T0 generation, DEGs were predominantly enriched in pathways related to drug metabolism, metabolism of exogenous substances by cytochrome P450 (CYP450), galactose metabolism, and starch and sucrose metabolism. In the T1 generation, DEGs were predominantly enriched in oxidative phosphorylation, drug metabolism, metabolism of exogenous substances by CYP450, and RNA polymerase pathways. In the T2 generation, DEGs were significantly enriched in oxidative phosphorylation, the metabolism of exogenous substances by CYP450, drug metabolism, and RNA polymerase pathways. Notably, the CYP450 metabolic processes of exogenous substances, drug metabolism, steroid hormone biosynthesis, and pentose and glucuronate interconversions were pathways commonly enriched in DEGs across the T0 to T2 generations.

In the drug metabolism pathway, 8, 28, and 30 DEGs were identified in the T0, T1, and T2 generations, respectively. Among them, *CBR1* (carbonyl reductase 1) (Log2FC Hap3T0: 1.71; Log2FC Hap3T1: 2.02; Log2FC Hap3T2: 2.61; *p* ≤ 0.05) and *UGTs* (UDP-glucuronosyltransferases) (Log2FC Hap3T0: 2.37; Log2FC Hap3T1: 2.84; Log2FC Hap3T2: 2.05; *p* ≤ 0.05) were consistently upregulated across all generations. The significant upregulation of these genes from T0 to T2 suggests that they may play a pivotal role in the adaptation of Hap3 *A. gossypii* to their new host environment, especially in drug metabolism and detoxification mechanisms.

### 3.4. Analysis of Differentially Expressed Genes Shared Between Hap1 and Hap3 A. gossypii After Host Transfer

**Figure 5:**
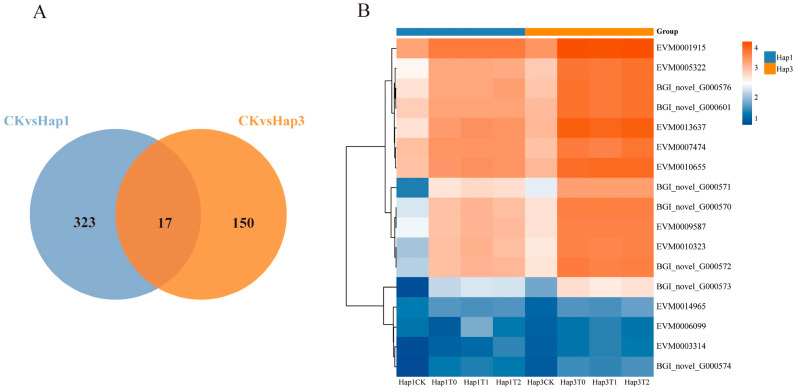
Joint Venn analysis of total DEGs (**A**); A-CKvsHap1: common DEGs in the T0-T2 generation after transferring Hap1 *A. gossypii* to zucchini; A-CKvsHap3: common DEGs in the T0-T2 generation after transferring Hap3 *A. gossypii* to zucchini; heatmap showing the abundance of DEGs shared between Hap1 and Hap3 groups (**B**), with orange cells indicating high expression and blue cells indicating low expression.

A total of 340 common DEGs were identified in the T0 to T2 generations of Hap1 *A. gossypii* following their transfer from cotton to zucchini. Similarly, 167 common DEGs were detected in the T0 to T2 generations of Hap3 *A. gossypii* after transfer from cucumber to zucchini. Comparative analyses revealed that 17 DEGs were shared between both groups, suggesting a possible conserved molecular mechanism underlying their adaptation to a new host environment (Figure 5A).

Figure 5B presents the results of the abundance correlation analysis of co-occurring DEGs between the T0 and T2 generations of Hap1 and Hap3 *A. gossypii* after transfer to zucchini. Among these shared DEGs, *CYP450 6a13*, encoded by EVM0006099, was the only gene that showed significant downregulation in both Hap1 and Hap3 *A. gossypii* from the T0 to T2 generations (−4.65 ≤ Log2FC Hap1T0-T2 ≤ −2.14; −2.55 ≤ Log2FC Hap3T0-T2 ≤ −1.86; *p* ≤ 0.05). In contrast, the expression of the remaining 16 genes was significantly upregulated.

Notably, several key enzyme genes were identified among the upregulated DEGs, including *Col-I*, encoded by EVM0001915 (1.53 ≤ Log2FC Hap1T0-T2 ≤ 1.87; 2.19 ≤ Log2FC Hap3T0-T2 ≤ 2.62; *p* ≤ 0.05), and *peroxidase*, encoded by EVM0003314 (1.00 ≤ Log2FC Hap1T0-T2 ≤ 2.19; 1.21 ≤ Log2FC Hap3T0-T2 ≤ 1.79; *p* ≤ 0.05).The expression levels of these key enzyme genes were significantly upregulated from T0 to T2 in both Hap1 and Hap3 *A. gossypii*, suggesting their potential roles in biological processes related to host adaptation.

## 4. Discussion

Many insects, especially aphids, are capable of surviving on a wide range of host plants and have evolved distinct biotypes [22]. The ability of insects to adapt to their specific hosts is primarily determined by genetic factors. However, their reproductive capacity and overall adaptation may also undergo significant changes in response to feeding on different host plants [23]. Previous studies have shown that the cotton-type Hap1 and cucumber-type Hap3 *A. gossypii*, which exhibit significant differentiation in host adaptability, are capable of switching hosts after being induced by the intermediate host, zucchini [20]. However, the genetic basis and molecular mechanisms underlying this zucchini-mediated host exchange remain unclear. In this study, changes in the body size of Hap1 and Hap3 *A. gossypii* following their transfer to zucchini were investigated, and the transcriptomes of the T0-T2 generations of these aphids were analyzed. The results demonstrated that body size was significantly affected by the zucchini host, and that changes in gene expression following host transfer were associated with insulin metabolism, detoxification mechanisms, nutrient absorption, and other biological processes. These contribute to a deeper understanding of host adaptation in *A. gossypii* and offer new perspectives on insect evolution and ecology.

The body size and fertility of *A. gossypii* are influenced by the nutritional conditions provided by their host plant [24]. Aphid size is dependent on the host plant, and shifts in host specialization are often accompanied by changes in individual size [25]. In this study, it was observed that after Hap1 and Hap3 *A. gossypii* were transferred to zucchini and reared for three generations, their body sizes increased significantly. This gradual increase suggests an enhanced ability to use zucchini as a host over successive generations. These findings align with previous research, which has demonstrated that body size typically reflects the adaptation of herbivorous insects to environmental conditions [26,27]. Aphids tend to decrease in size in deteriorating environments and, conversely, increase in size when environmental conditions are favorable [28,29].

The insulin signaling pathway plays a central role in regulating several aspects of insect biology, including metabolic homeostasis, growth, reproduction, development, and longevity [30]. In this study, DEGs associated with the insulin secretion pathway were significantly enriched in the T0-T2 generations of Hap1 *A. gossypii* following their transfer to zucchini. This finding is consistent with previous research, which has shown that aphids encountering a new host plant may face stressors such as changes in nutrient composition and plant defense responses. To adapt, aphids regulate their metabolism and physiological state through adjustments in insulin secretion [31]. Additionally, the expression of *IGF2*, a key gene associated with the insulin secretion pathway, was found to be significantly upregulated in the T0-T2 generations of Hap1 *A. gossypii* after host transfer. *IGF-2* plays a critical role in preventing cell death by promoting cell growth and proliferation [32]. The observed upregulation of *IGF-2* expression in Hap1 *A. gossypii* may represent an adaptive strategy that facilitates cell division and tissue growth, ultimately enhancing their survival and adaptation to the new host.

After transitioning to a new host plant, aphids are subjected to selective pressures by different plant species, leading to corresponding adjustments in their metabolism, nervous system, and detoxification enzyme system. The utilization of detoxification enzymes serves as an efficient strategy for aphids, allowing them to mitigate the toxicity of plant secondary metabolites while reducing energy expenditure and survival costs [33]. In this study, it was found that after Hap3 *A. gossypii* were transferred to zucchini hosts, the DEGs in the T0-T2 generations were significantly enriched in the CYP450-mediated metabolism of exogenous substances and drug metabolism pathways. This finding suggests that zucchini hosts may have imposed selective pressure, prompting adjustments in the aphid’s detoxification enzyme system to improve its ability to adapt and reproduce in the new environment. Insects facing stress from endogenous or exogenous compounds rely on detoxification metabolic pathways to protect themselves from harm [34]. Additionally, this study identified two detoxification enzyme genes with significantly up-regulated expression in the drug metabolism pathway: *CBR1* and *UGTs*. *CBR1*, as an NADP-dependent enzyme with detoxification functions, catalyzes the conversion of carbonyl-containing compounds [35]. *UGTs* facilitate the conjugation of diverse lipophilic small molecules with sugar substrates, resulting in the production of water-soluble glycoside derivatives that enable detoxification and metabolic clearance [36]. These enzymes play a crucial role in the detoxification of plant secondary metabolites in insects [37]. When transferred to zucchini hosts, *A. gossypii* are likely exposed to a new chemical environment containing plant secondary metabolites and potentially toxic substances (e.g., cucurbitacin, triterpenoids). The observed upregulation of *CBR1* and *UGTs* gene expression may facilitate the detoxification and more efficient metabolism of these newly encountered compounds, thereby enhancing the aphids’ survival and adaptation to zucchini.

The specific expression of certain genes following host transfer may contribute significantly to the differentiation of aphid biotypes [38]. In this study, transcriptome analysis identified seventeen genes that exhibited consistent differential expression across the T0 to T2 generations of Hap1 and Hap3 *A. gossypii* after transfer to zucchini hosts. Notably, the expression of *Col-I* and *peroxidase* was significantly upregulated—by more than twofold—in both Hap1 and Hap3 aphids. *Col-I*, an essential component of the insect exoskeleton and extracellular matrix, provides structural support and protection [39]. The upregulation of *Col-I* gene expression may reinforce the strength and stability of the *A. gossypii* exoskeleton, enabling better resistance to the physical defense mechanisms of zucchini. This adaptation may improve the aphid’s ability to securely attach and feed on the new host. In addition to physical challenges, aphids transferred to zucchini may encounter oxidative stress due to plant-induced defense responses. Upon aphid infestation, zucchini plants initiate defense mechanisms that produce large quantities of reactive oxygen species (ROS), which are toxic to aphids [40]. Peroxidase is an important antioxidant enzyme that catalyzes the breakdown of ROS, including hydrogen peroxide (H_2_O_2_), thereby mitigating oxidative stress-induced cellular damage in aphids. The observed upregulation of *peroxidase* gene expression in Hap1 and Hap3 aphids may facilitate more efficient ROS scavenging, maintain intracellular redox balance, and enhance resilience in the new host environment. In this study, a significant downregulation of *CYP450 6a13* gene expression was observed in the T0 to T2 generations of Hap1 and Hap3 aphids after transfer to zucchini hosts. This downregulation may be attributed to the absence or reduction in the specific secondary metabolites present in the original host, which may have acted as signaling molecules to activate *CYP450 6a13* expression. In the new host environment, the suppression of this gene could serve as an evolutionary adjustment, allowing for improved adaptation to zucchini. Consistent with previous findings, the expression of detoxification genes in insects varies significantly in response to host plant secondary metabolite stress, climatic stress, and pesticide exposure [41].

In summary, after transitioning to zucchini hosts, Hap1 and Hap3 *A. gossypii* may overcome physical and chemical barriers by inducing changes in key genes and metabolic pathways in response to host-imposed stress. These adaptive changes may, in turn, influence host range expansion and specialization phenotypes. Future studies should focus on further investigating the DEGs identified in this study to identify their roles in *A. gossypii* biotype differentiation and evolution.

## 5. Conclusions

The introduction of mixed cropping patterns can enhance land use efficiency but may also alter insect feeding behaviors, potentially increasing pest damage. The *A. gossypii*, a host-specialized insect, modifies its host preference after feeding on a specific plant. This study revealed that Hap1 and Hap3 *A. gossypii* significantly increased in body size after being transferred to zucchini hosts. Transcriptome analysis showed that these aphids significantly upregulated genes related to insulin secretion and detoxification metabolism following transfer to zucchini. These findings provide novel insights into insect–host plant interactions and their adaptive mechanisms, offering valuable perspectives for the development of more targeted and effective pest management measures, which are critical for sustainable agricultural practices.

## Data Availability

The original contributions presented in this study are included in the article. Further inquiries can be directed to the corresponding authors.
